# Decomposing the Temporal Signature of Nitrogen Dioxide Declines during the COVID-19 Pandemic in UK Urban Areas

**DOI:** 10.1007/s12061-022-09438-2

**Published:** 2022-04-12

**Authors:** Alessia Calafiore, Jacob L. Macdonald, Alex Singleton

**Affiliations:** 1 Geography and Planning, University of Liverpool, Chatham St, Liverpool, L69 7ZT UK; 2 Department of Urban Studies and Planning, University of Sheffield, Western Bank, Sheffield, S10 2TN UK

**Keywords:** COVID-19, Air Pollution, Human Mobility

## Abstract

On March 23, 2020, a national lockdown was imposed in the UK to limit interpersonal contact and the spread of COVID-19. Human mobility patterns were drastically adjusted as individuals complied with stay-at-home orders, changed their working patterns, and moved increasingly in the proximity of their home. Such behavioural changes brought about many spillover impacts, among which the sharp and immediate reduction in the concentration of nitrogen-based pollutants throughout the country. This work explores the extent to which urban Nitrogen Dioxide (NO_2_) concentration responds to changes in human behaviour, in particular human mobility patterns and commuting. We model the dynamic and responsive change in NO_2_ concentration in the period directly following national lockdown and respective opening orders. Using the national urban air quality monitoring network we generate a synthetic NO_2_ concentration series built from a time series of historic data to compare expected modelled trends to the actual observed patterns in 2020. A series of pre- and post-estimators are modelled to understand the scale of concentration responsiveness to human activity and varying ability of areas across the UK to comply with the lockdown closing and response to openings. Specifically, these are linked to workday commuting times and observed patterns of human mobility change obtained from Google mobility reports. We find a strong and robust co-movement of air pollution concentration and work-related mobility – concentrations of NO_2_ during typical weekday commuting hours saw a higher relative drop, moving in tandem with patterns of human mobility around workplaces over the course of lockdowns and openings. While NO_2_ concentrations remained relatively low around the time of reopening, particularly during commuting hours, there is a relatively fast responsiveness rate to concentrations increasing quickly in line with human activity. With one of the key Government advice for workers to take staggered transportation into work and lessen the burden of rush hours and adopting more flexible work-home arrangements, our results would suggest that reductions in NO_2_ in urban areas are particularly responsive to broader human patterns and dynamics over time as we transitioned towards new working routines.

## Introduction

Air quality and pollution, especially in the urban context, are closely linked to human activity and movement. Whether from transportation, energy consumption or industrial processes, the spatial distribution of air pollutants is intimately related to the spatial distribution and temporal signature of individual and economic activity. This is particularly true for nitrogen-based pollutants which are linked to combustion and transportation in urban areas and especially responsive to changes in the level of human activities (AQEG 2004; DEFRA 2020).

Over the course of the COVID-19 pandemic to assist in stopping the virus spread, most government bodies imposed lockdown and shelter-in-place policies. In the UK the first such national policy was introduced on March 23, 2020, and led to an unprecedented shift in how day-to-day human activity is carried out.^1^ With citizens across the country, and the globe, urged to stay at home, regular workday mobility patterns were replaced with reduced, staggered movement and social distancing. This led to an almost immediate shift to increasingly local movement around residences and less around workplaces, patterns still lingering as varying portions of the economy continue to work from home.

Reduction trends in pollution have been seen across the world in tandem as emissions concentrations react to fast changing and varying degrees of national and regional lockdowns and enforcement. While varying degrees of national, regional and local lockdowns and openings have occurred in different contexts across the world, preliminary estimates point to global reductions across all pollution categories. Estimates of population-weighted global ground-level NO_2_ reductions are on average 60% with a 31% decrease in particulate matter (Venter et al., 2020), and peak reductions in CO2 emissions of 26% (Le Quéré et al., 2020).

Across different regions and cities however, this reduction in pollution can vary in scale and intensity with estimates of around 30% average drop in NO_2_ in areas of Wuhan, the USA and Europe (Muhammad et al., 2020; Berman & Ebisu, 2020; Liu et al., 2020), while reductions in Delhi have been estimated to exceed 50% to 60%, primarily linked to traffic (Mahato et al., 2020) and up to 78% in Mumbai (Kumari and Toshniwal, 2020). Across cities in China, large-scale lockdowns have led to reductions of NO_2_ emissions in the range of 24.67% compared to the relative decrease of human mobility which dropped by 69.58% (Bao & Zhang, 2020). In the US a similar average decline in NO_2_ of 25.5% is observed in counties implementing early lock-downs (Berman & Ebisu, 2020).

In the UK, mean reduction in NO_2_ emissions of around 30–50% were observed during the lock-down, particularly at roadsides (AQEG 2020; Ropkins and Tate, 2021). Along with the widely reported decrease in NO_2_, CO_2_ and particulate matter, it is important to note the highly complex nature of different pollutants with findings of increased O_3_ levels during the lock-down (AQEG 2020; Higham et al., 2020; Collivignarelli et al., 2020; Sicard et al., 2020), and SO2 in the UK (Higham et al., 2020).

This research leverages the high resolution of air pollution data to explore NO_2_ concentration responsiveness in the face of national lockdown and openings, compared to what would have been the status quo. We implement a difference-in-difference approach to simulate a quasi-experimental design to study observed concentration impacts against a controlled sample (Chen et al., 2020). We estimate a synthetic control data series conditional on temporal dynamics and meteorological conditions, subsequently used to build a longitudinal panel database and explore the sequential average daily area reduction in pollution in the immediate and five week adjustment period following respective lockdown and opening interventions. Specifically, we estimate daily station level synthetic NO_2_ under a series of autoregressive integrated moving average (ARIMA) predictions on five years of respective pollution and meteorological trends. The high dimension of our data and estimation procedure allows us to consider and trace out extremely detailed immediate and short-run announcement effects linked to particular changes in mobility patterns.

While our results confirm trends seen in other studies (AQEG 2020; Ropkins and Tate, 2021), we contribute to the existing body of knowledge by further investigating how the varying ability to comply with mobility restrictions imposed by the government across the UK resulted in different reductions in NO_2_. To do this we combined air pollution with mobility data provided by Google, which categorised people’s spatial behaviour change over the course of the pandemic in terms of visits to different place types (i.e. workplace, home, grocery shops). For example shifts towards home-based) work patterns are one margin through which pollution dynamics may be influenced, with time saving and a potential reduction in traffic congestion among the main points stressed by the early advocates of remote working (Kitamura et al., 1991; Olson, 1983).

However, works in transportation studies have shown conflicting results. Although reductions in the number and length of commuting trips is reported in some of the earliest studies (Kitamura et al., 1991), more recent research shows that the link between home-based work and travel reduction is not so apparent (de Abreu e Silva and Melo 2018; Budnitz et al. 2020) and time saving seems not to be a major pull factor (Bailey & Kurland, 2002).

This work contributes to understanding the extent to which changes to human activities, and in particular new everyday work patterns, have impacted combustion pollution concentration levels in the immediate and short run. This is an important first step in better understanding how different areas, with vastly different propensities to work from home and ability to comply with stay-at-home orders, have been differently impacted by these responsive changes to pollution dynamics and therefore resulting in people experiencing unequal exposure to air pollution where they live.

## Data and Study Region

High temporal frequency pollution monitoring and meteorological data are obtained from the UK Government Department for Environment, Food and Rural Affairs (DEFRA). Various monitoring networks exist, each targeting different types of pollutants in different areas. We focus on capturing the high-frequency transition of daily patterns as they adjust under new lockdown and easing periods in urban areas, and the role that this transition has on pollution concentrations. Therefore, we use the Automatic Urban and Rural Network (AURN)^2^ measuring hourly levels of Nitrogen Dioxide (NO_2_) concentrations measured in µg/m^3^, distinguishing between two sets of stations: Urban Background capturing more ambient levels and Urban Traffic targeted at roadside concentration monitoring.

**Fig. 1:**
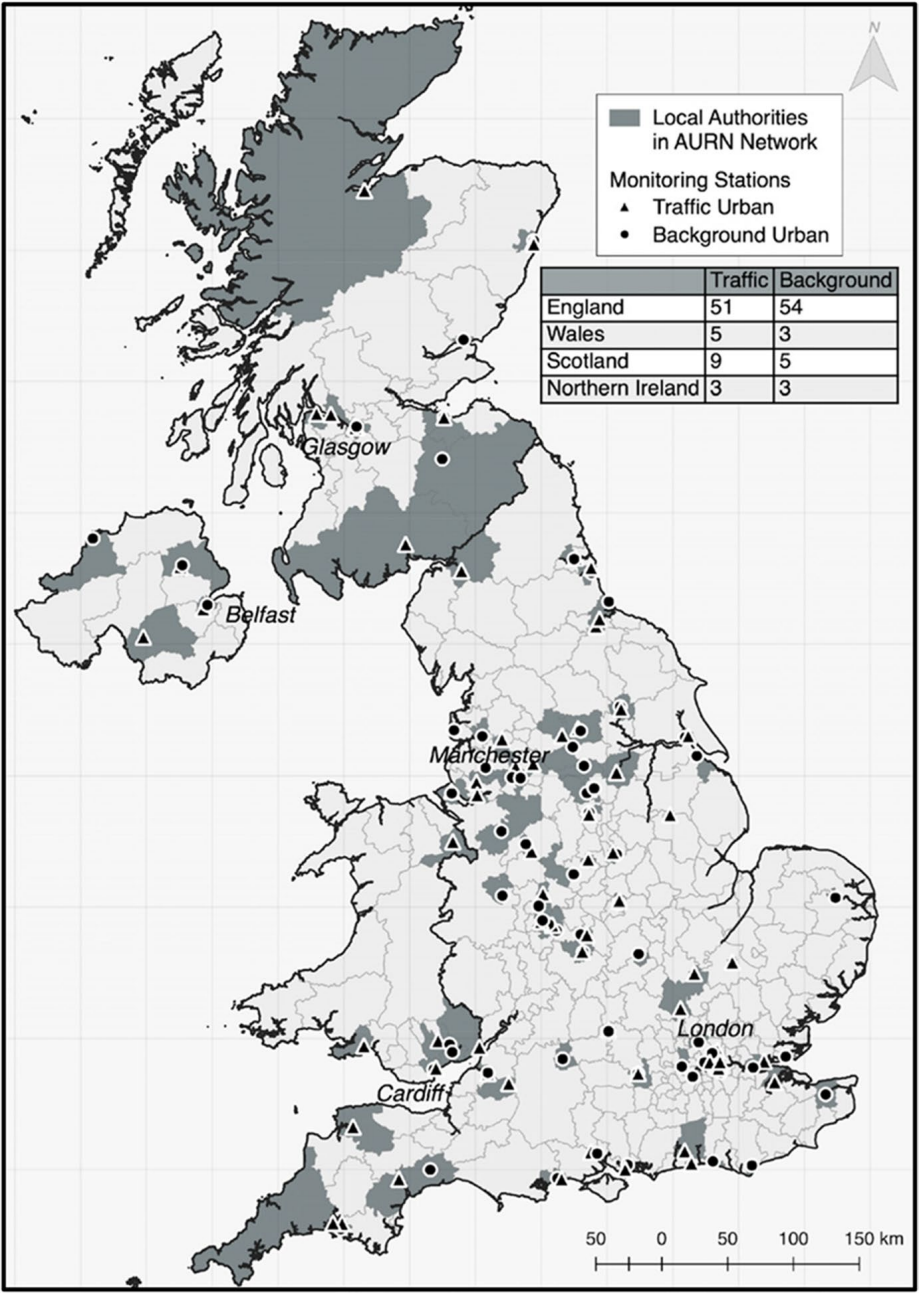
AURN Pollution Monitoring Network UK

AURN network monitoring stations are categorised according to the primary type (or source) of urban pollution located in continuously built-up areas. Pollution at traffic sites is determined predominantly by emissions from nearby vehicles and representative of air quality in major city junctures, while background sites represent areas and pollution levels not determined particularly by any single source or street but rather the integrated contribution from all sources upwind. In total the network comprises 133 stations with 65 Background and 68 Traffic. Figure 1 shows the distribution of station types across the UK, highlighting respective Local Authority administrative boundaries which serve as our boundary definitions.

With a few exceptions, there is a general one-to-one correspondence between Local Authority areas and the presence of a Background station, a Traffic station, or both. Separate analyses are conducted on the traffic-based and background-based pollution measures for a variety of reasons. Firstly, since the observational unit defaults to the Local Authority we risk over-representing a location by including multiple same-source monitors in a local area. In the case where a Local Authority has two or more of the same monitoring source, an average value is taken for that region’s traffic or background levels. There are only five Local Authorities (Stockton-on-Tees, Sheffield, Birmingham, Aberdeen City and Glasgow City) with more than a unique type, and in these areas both of the same source monitoring stations are located in close proximity to each other.^3^

We further keep traffic and background pollution isolated as opposed to pooled given the significant difference in their levels and patterns. A t-test on the values traffic and background pollution measured within the same locality indicate significantly higher average traffic levels with a mean value of 3.17 relative to the 2.74 mean of background levels and $t=-84.62$. Thus, the location observational units used are based on 64 unique Local Authorities with Traffic monitoring stations and 63 unique Local Authorities with Background monitoring stations respectively, of which 31 have both types of stations.

**Fig. 2:**
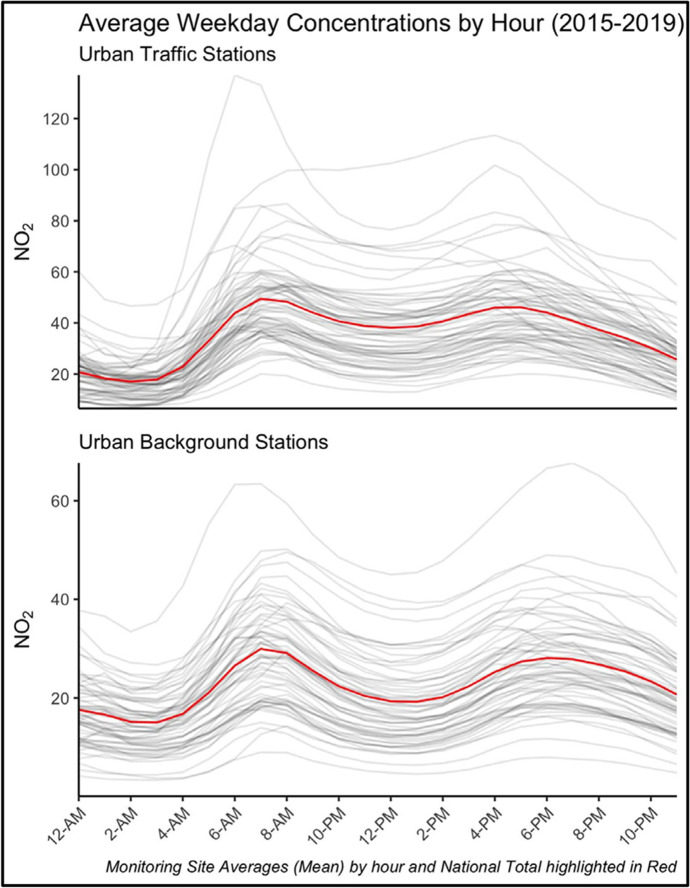
Hourly Pollution Trend Aggregates

Hourly pollution can be aggregated in several ways to explore varying dynamics. To align with pre-pandemic workday temporal signatures, we explore not only the daily median value of NO_2_ concentrations, but further distinguishing between the median value of pollution occurring during peak rush-hours and working hours.^4^ Figure 2 shows the weekday average daily temporal signature of pollution over time for Local Authorities compared against the national average. These patterns highlight peak rush-hour times and concentration levels over the 24-h cycle, and are more prominent in high traffic areas. With this as our baseline temporal workday signature, we leverage the high frequency hourly pollution levels to explore not only the reduction in daily concentrations, but whether they are linked to average changes over particular key times of the day.

**Fig. 3:**
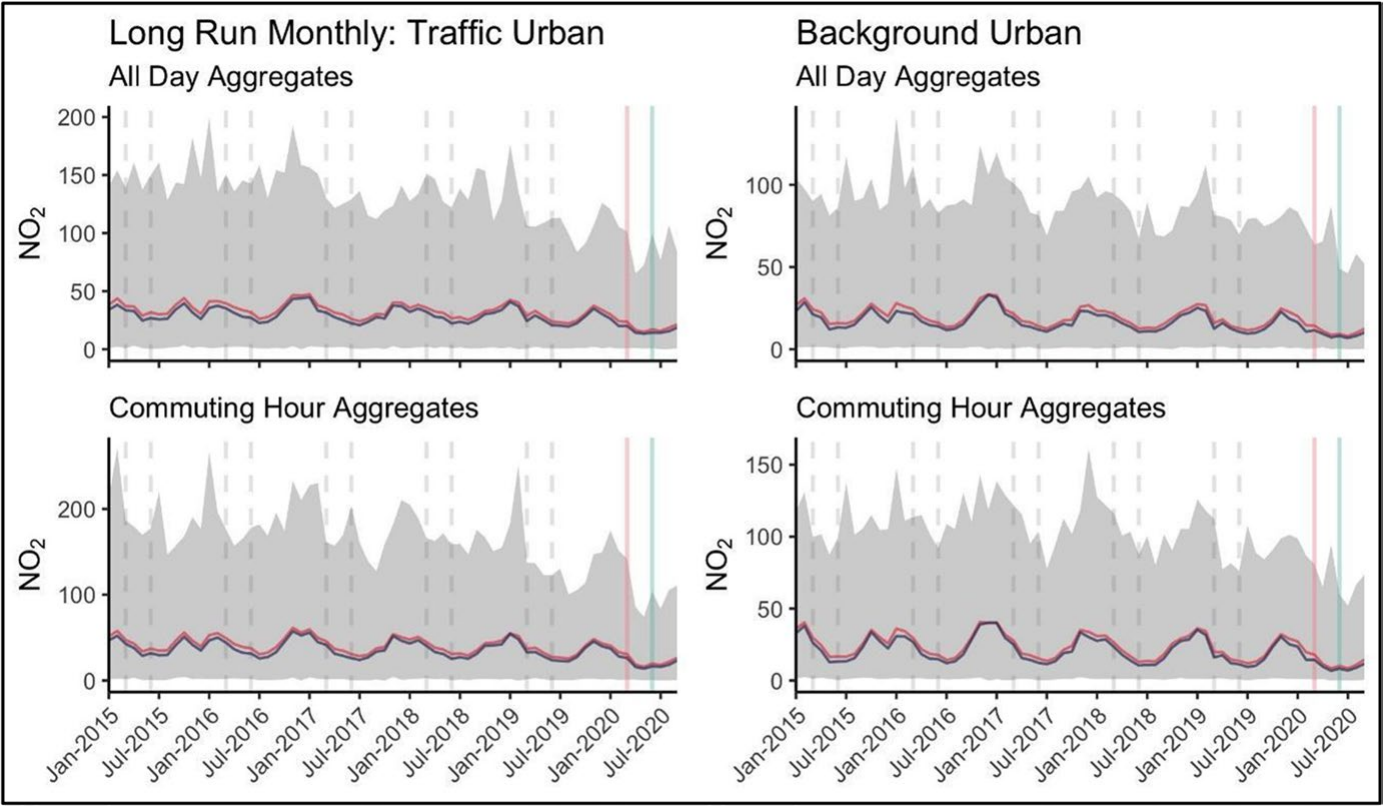
Long Run Pollution Trends, Monthly Aggregates: Min/Max (shaded); Mean (red), Median (Blue). The lockdown/ easing period is marked by the red and green vertical lines; dashed vertical lines highlight the same period in previous years

Decomposing longer term trends, Fig. 3 plots the distribution of daily NO_2_ dynamics since 2015.^5^ These plots show the maximum and minimum national pollution level along with mean and median values. The data is sliced in different ways, firstly by traffic and background urban stations, and further by time signature including the median daily value for the entire 24-h period and the median value observed during rush-hours (identified in Fig. 2 as 7AM – 9AM and 4PM – 6PM).

While longer term NO_2_ reductions are less evident in only looking at the average value over time, we see particular declines in peak maximum pollution levels in recent years. Traffic-based monitoring levels show some evidence of general decline over the past five years, most notable in commuting hour concentration levels.

Important is the seasonal patterns which can be observed over the multi-year series. The smoothed average and median trends highlight emission peaks occurring in December and January of the year, declining into the summer. This nationally aggregate seasonal decrease observed in the data during the summer months is especially important to consider in trying to discern specific impacts from lockdown and opening measures versus more general and recurring temporal dynamics. These plots reveal the magnitude of difference between roadside and ambient concentration levels and dynamics around the lockdown period and comparable times in previous years. These longer term temporal dynamics and patterns in the data are important to consider when building the appropriate counterfactual data series to be used in our empirical application.

**Fig. 4:**
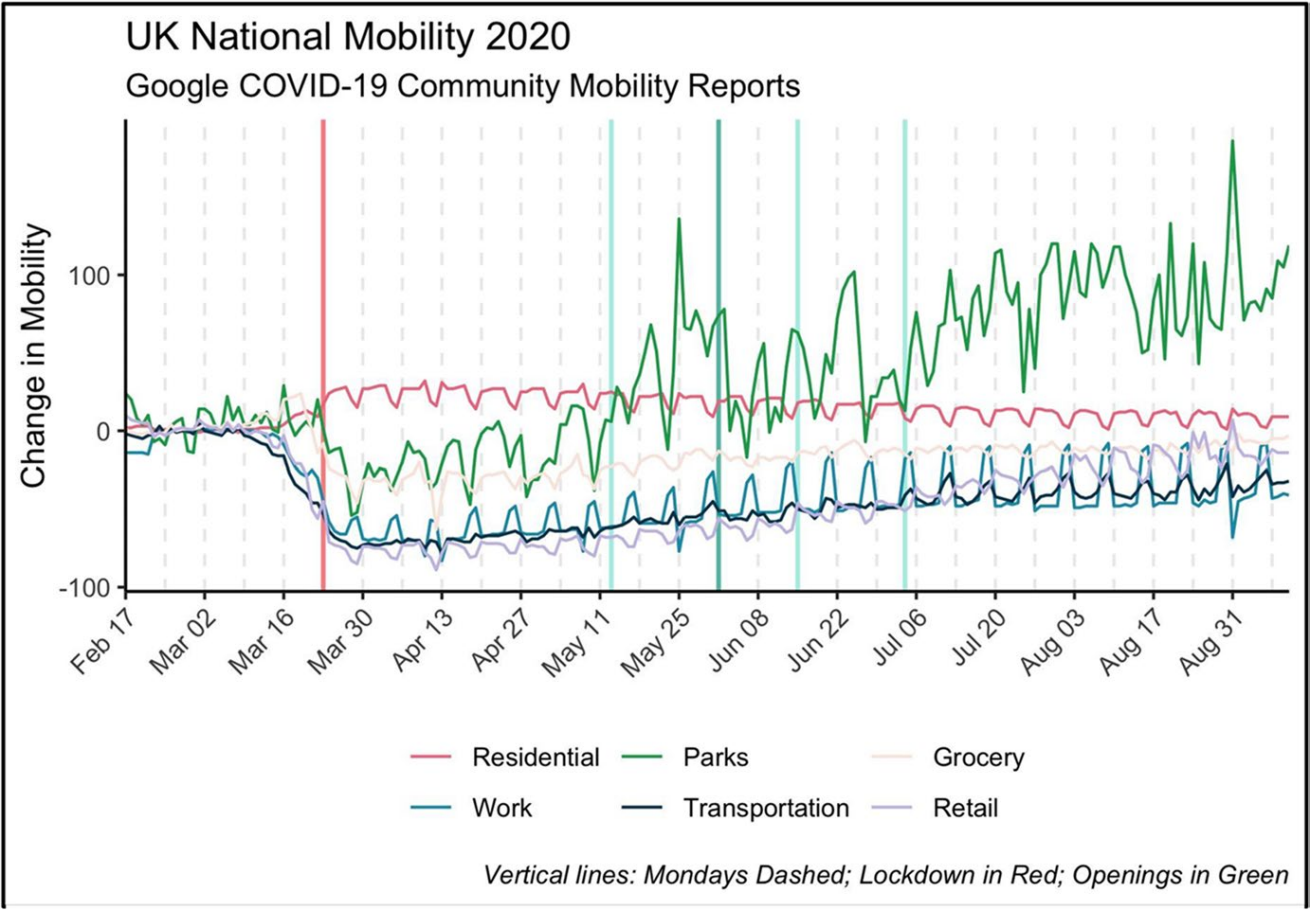
UK National Mobility Patterns

With the correspondence between the pollution monitoring network and Local Authorities we can further exploit any potential variation in Local Authority characteristics. If areas have different underlying mechanisms and dynamics through which pollution is impacted, auxiliary data at this level can be used to explore these variations. While time-invariant characteristics of the area will drop out in the difference-in-difference estimation, we use daily Local Authority mobility changes as measured by the Google Community Mobility Reports.^6^ This measure is the relative deviation from a baseline in visits to places of interest across six different category groups: Retail and Recreation; Grocery and Pharmacy; Parks; Transportation; Workplaces; and Residential (Fig. 4). The baseline is calculated as the median value of visits for the corresponding day of week during the 5-week period from January 3 to February 6, 2020.

These measures of mobility are a good proxy to determine whether expected changes in mobility were observed (Adams, 2020). Such measures are extremely correlated with pollution emissions and the timing of the lockdown and respective openings, and we see clear patterns following the lockdown with mirroring images suggesting significant increases in residential mobility and drops in more non-residential areas.

## Methods

This work decomposes estimates for the reduction in NO_2_ concentration levels conditional on the spatio-temporal signature of change and how they align with pre-pandemic workday patterns. While recent changes to human activities are likely to have a range of influences on any number of pollutants, we focus on NO_2_ given its direct relation with dynamic changes in cities such as road traffic and human mobility patterns.

On March 23, 2020, and in response to the increasing spread of COVID-19, the UK government implemented the first stage of a national lockdown strategy during which time mass stay-at-home order were enforced, workplaces and retail areas shuttered, and residents permitted to leave their dwellings for limited exercise or emergency situations. During this time, while the largest portions of the national workforce worked from home, a small subset of industries and key occupations continued to operate at limited capacity. Key-workers, including health and social care, food distribution networks, limited educational institutions and child-care, government and utility workers remained in many areas the only individuals commuting to and from home and work – where secure to do so.

Following this lockdown, varying degrees of national-level opening up announcements were made effectively easing the regulations. These announcements were cumulative and referred to certain restrictions being relaxed and official opening of workplaces and non-essential businesses.^7^ The first of these *easings* occurred on May 13, 2020, when the public was encouraged to *stay-alert* and return to work if a job was not able to be done from home (e.g. those in construction, manufacturing and primary industries). Following this, it was announced on June 1 that limited outdoor interaction was possible between different household bubbles with non-essential shops allowed to reopen on June 15, 2020. The final major national announcement regarding the reopening phase was on July 4 when restaurants, pubs and cafes were further allowed to open.^8^

Using the first lockdown and opening dates as benchmarks, we analyse the impact of the subsequent behavioural changes to concentration of NO_2_ in UK cities. First, we explore observed temporal shifts in the distribution of NO_2_ concentration to have an overview of potential relationships with work-related activities. While the data exploration provides an overview of such relationships, we then estimate gaps between observed 2020 pollution around these dates and synthetic estimate NO_2_ series, further accounting for temporal and meteorological trends. Finally, we decompose these estimates according to human mobility changes recorded from Google and published via Google mobility reports. This allows us to shed light more specifically on which human activities have influenced the dynamics and responsiveness of NO_2_ concentrations. Using these high frequency treatment–control pairs with daily variation allows us to explore the immediate and adjusting short-run influences in the days and weeks following the announced changes.

### Synthetic Control Data

Estimating the impact of lockdown and easing measures on combustion-based pollution requires a valid and robust counterfactual pollution dynamic against which to compare. We are interested in benchmarking the impact of the lockdown and easing on pollution levels under the alternative assumption of no lockdown and where NO_2_ emission levels would have continued along their expected trend. Comparing current pollution dynamics against a counterfactual not truly representative of the alternative risks over- or under-estimating impact magnitudes. We build our daily alternative pollution levels from predicting on the time series of each individual location using an estimated ARIMA model on respective data, and covariates, from 2015 to 2020. The ARIMA model is a widely used linear method for time-series analysis (Shumway & Stoffer, 2017), frequently applied to forecast air pollution (Nieto et al., 2018; Wang et al., 2017). The most general form of the model, which is fit individually to each local monitoring time series, is as follows:

1 $$lnln\left(y_t\right)=\sum_{i=1}^{p}\phi_{i} lnln\left(y_{t-i}\right)+\sum_{k=1}^{r}\beta_{k} x_{tk}+\varepsilon_{t}+\sum_{j=1}^{q} \theta_{j} \varepsilon_{t-j}$$

where the daily emission level ${y}_{t}$ for a given area is modelled conditional on the time series of lagged historic levels ${y}_{t-i}$, a vector of $k$ exogenous covariates ${x}_{tk}$ including wind speed, direction, temperature and a dummy variable indicating weekend status, and a series of autocorrelated error terms ${\varepsilon }_{t-j}$.

The choice of $p$ and $q$ which define the strength of temporal spillovers are chosen in an automated process across all areas respectively. A cross validated approach iteratively fits a series of model specifications varying these parameters and selects $p$ and $q$ to minimise the AIC value. In the above specification ${y}_{t}$ represents the stationary version of the pollutant time series. The integrated component of the ARIMA model accounts for non-stationarity and seasonality patterns in the data, further testing and parameterizing these influences.

Systematically for every location we use the time series of five years historical pollution and meteorological data to estimate respective models to capture traditional regional pollution dynamics up until the ongoing COVID-19 changes began. The models are estimated on the (log) daily median NO_2_ levels from 2015 until the first known reported case of COVID-19 in the UK on January 31, 2020, before which there was little mention of the virus in local media.^9^

It is from this parameterized model, trained in the *non-COVID* world, that we predict synthetic control pollution time series for each location. Using observed pollution levels and meteorological conditions from February 1 onwards, we predict the expected value of emission levels. The underlying idea is that our synthetic data is representative of the overall trends and expected behaviour in pollution concentration had NO_2_ dynamics over the past five years propagated forward as expected.

The benefit of this automated sequential ARIMA process applied across each monitoring station is in generating a very high temporal resolution of pollution data which is representative of the expected daily pattern for February, 2020, and beyond. This allows us to select comparable days from which to evaluate deviations and adjusts for longer term seasonal or cyclical trends. When comparing a summertime lockdown impact on pollution, it is important these sub-year seasonal shifts are accounted for. Estimates comparing impacts over dis-similar time periods, or only using a comparison of same-time 2019 pollution data may overestimate the impact of lockdown and openings if we are unable to account for the pre-existing temporal trends and dynamics in each individual monitoring level time series.

**Fig. 5:**
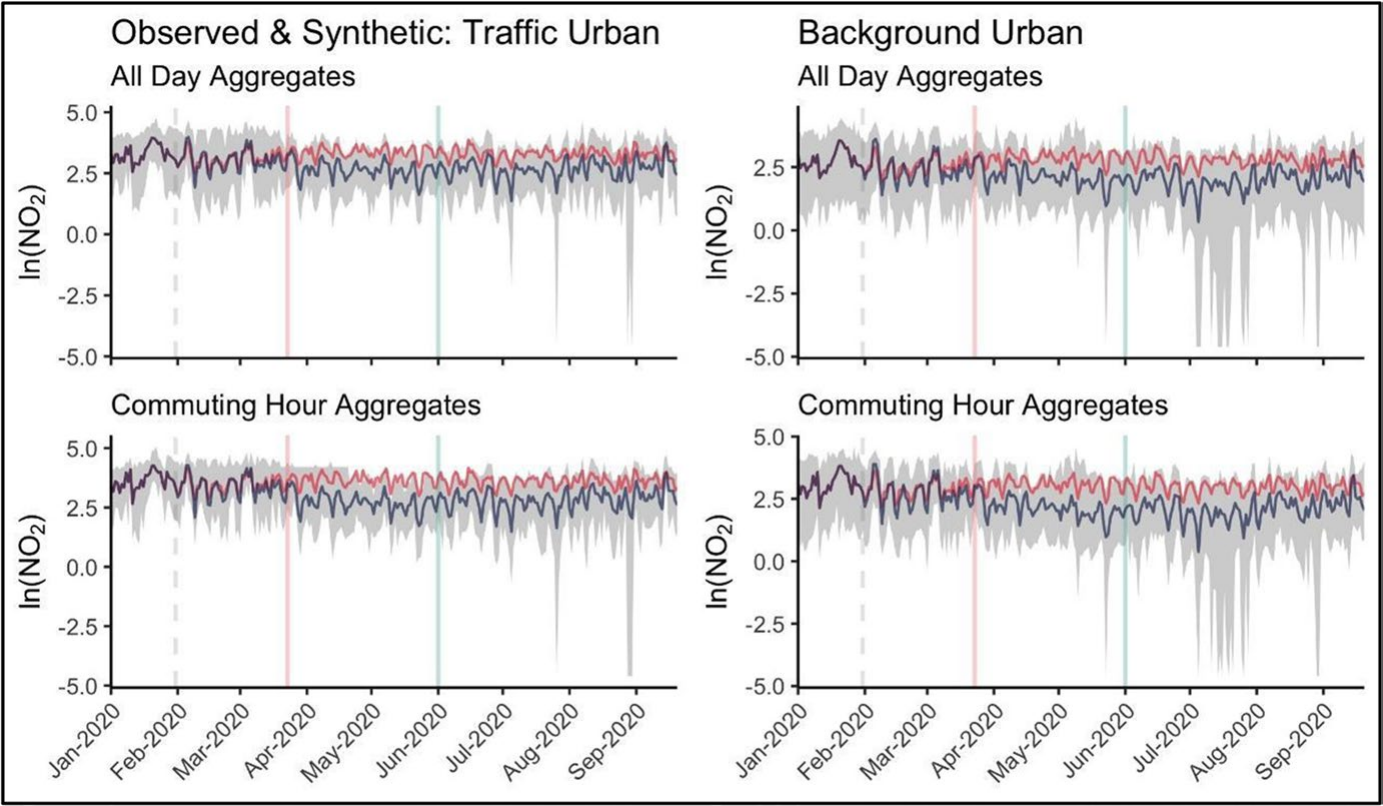
Synthetic Pollution Estimation. Min/Max (shaded); Observed Mean (Blue); Synthetic Mean (Red)

Our interest is in evaluating the average wedge between the (treated) observed current pollution trends across the country and the synthetic forecasted values they would have taken had no lockdown restrictions been enforced. Figure 5 gives the average national version of this effect taking respective time series predictions from each monitoring station and aggregating to a national trend. This plot highlights one of the crucial assumptions upon which impact estimates are built, namely that the pre-intervention (lockdown and opening) movements in the treated (observed) and control (synthetic) pollution series follow similar patterns and trends.

### Difference-in-Difference Impact Estimation

We build a longitudinal database of daily median NO_2_ pollution levels spanning the traffic monitoring in $l=64$ locations and background monitoring in $l=63$ locations. For all areas, we have both a treated and control data series identifying observed concentration and synthetically estimated concentration. Subsets of commuting hour dynamics are also extracted to explore specifically how these impacts relate to pre-treatment workday habits and patterns.

The most general form of the fixed effects panel data model estimated is given as follows. This generic specification nests multiple models varying in terms of the lockdown or easing intervention of interest, and further considering full day and commuting hours impacts.

2 $$lnln\left(y_{lt}\right)=\alpha_{0}+{\beta_{0}P}_{t}+{\beta_{1}T}_l+\beta_{2}\left(P_{t}\cdot T_{l}\right)+\beta_{3} X_{lt}+u_{lt}$$

One of the key assumptions upon which this method of estimation is based is that of parallel trends in the two comparable data series prior to the treatment intervention. From the aggregate in Fig. 5, both trends have the same dynamics prior to the lockdown intervention with the synthetic data then diverging to represent a comparable path were it not for the intervention. From the period between January 31, 2020, to the lockdown both the predicted series and the observed series are matched with relative accuracy. This persists until around March 23, 2020, where a clear downward shift in real-world pollution levels are observed.^10^

We focus on the lockdown of March 23, 2020, and one of four easings, June 1, 2020, to compare closing and opening effects.^11^ The benchmark difference-in-difference model is estimated sequentially on the treatment–control pairs for increasing time spans surrounding the respective lockdown and easing announcement of interest. This allows us to estimate the impact of the lockdown at daily intervals for the immediate days following up to five weeks. Exploring the daily adjusting nature of pollution concentration demonstrates the clear responsiveness to changes in human activity.

To further tie the estimated impacts to changes in the broader temporal signature and behavioural shifts introduced during this time period, each model is estimated in turn on varying data subsets. First we compare pollution levels for full daily aggregates versus commuting hour aggregates. We then condition the models by estimating in turn on subsets of Local Authorities correspondingly with above median and below median mobility changes after lockdowns and openings to explore how these areas may differ in their dynamic NO2 impacts.

## Results

### Decomposing Temporal Signatures of Average Emission Reductions

A series of estimates are obtained sequentially exploring the impact of the first lockdown (March 23, 2020) and opening (June 1, 2020) on urban background and traffic NO_2_ dynamics. The estimated impacts control for the average difference between the observed and synthetic predicted pollution levels corresponding to ${\beta }_{1}$ from Eq. 2 (Obs-vs-Pre). The interaction component with the respective lockdown or easing announcement ${\beta }_{2}$ estimates the treatment effect of such policies on current (treated) pollution levels (Diff-in-Diff). In looking at both parameter estimates jointly we are able to decompose global average emission reductions into the effects due to differences between the observed (treated) and predicted (control) levels and the lockdown or easing specific components at daily intervals. Selected parameter estimates on these impacts and up to five weeks following the respective announcements are presented in Table 1.^12^

**Table 1:** Difference-in-Difference Impact Estimates (Robust SE)

	Pre/Post Lockdown (March 23)					Pre/Post Opening (June 1)				
	*3 Days*	*5 Days*	*1 Week*	*3 Weeks*	*5 Weeks*	*3 Days*	*5 Days*	*1 Week*	*3 Weeks*	*5 Weeks*
*Daily Traffic Pollution*	**MODEL 1**					**MODEL 2**				
Obs-vs-Pre:${\beta }_{1}$	-0.39134^***^	-0.31497^***^	-0.29152^***^	-0.12275^***^	-0.11095^***^	-0.68688^***^	-0.71058^***^	-0.80610^***^	-0.86635^***^	-0.82002^***^
	*(0.06833)*	*(0.05392)*	*(0.04631)*	*(0.02796)*	*(0.02221)*	*(0.06892)*	*(0.05313)*	*(0.04557)*	*(0.02784)*	*(0.02130)*
Diff-in-Diff.:${\beta }_{2}$	0.24591^***^	0.00825	-0.18480^***^	-0.47611^***^	-0.54635^***^	0.05637	0.04097	0.07388	0.19428^***^	0.14563^***^
	*(0.08607)*	*(0.07077)*	*(0.06383)*	*(0.03908)*	*(0.03068)*	*(0.09294)*	*(0.07437)*	*(0.06453)*	*(0.03968)*	*(0.03168)*
*Adjusted R*^*2*^	*0.41301*	*0.43431*	*0.44226*	*0.4131*	*0.39889*	*0.42749*	*0.48107*	*0.49898*	*0.50989*	*0.50425*
*Commuting Traffic Pollution*	**MODEL 3**					**MODEL 4**				
Obs-vs-Pre:${\beta }_{1}$	-0.46292^***^	-0.39139^***^	-0.34510^***^	-0.17257^***^	-0.15923^***^	-0.84582^***^	-0.88965^***^	-1.00798^***^	-1.09809^***^	-1.05345^***^
	*(0.07269)*	*(0.05478)*	*(0.04592)*	*(0.02696)*	*(0.02117)*	*(0.07527)*	*(0.05664)*	*(0.04803)*	*(0.02844)*	*(0.02180)*
Diff-in-Diff.:${\beta }_{2}$	0.17952^**^	-0.04332	-0.25511^***^	-0.59943^***^	-0.68086^***^	0.03325	0.03307	0.08755	0.24659^***^	0.19472^***^
	*(0.09064)*	*(0.07229)*	*(0.06447)*	*(0.03982)*	*(0.03085)*	*(0.09807)*	*(0.07722)*	*(0.06679)*	*(0.03994)*	*(0.03178)*
*Adjusted R*^*2*^	*0.42879*	*0.47994*	*0.5043*	*0.46414*	*0.46081*	*0.49746*	*0.54766*	*0.56699*	*0.58953*	*0.58035*
Observations	406	638	870	2,494	4,118	406	638	870	2,494	4,118
*Daily Background Pollution*	**MODEL 5**					**MODEL 6**				
Obs-vs-Pre:${\beta }_{1}$	-0.42486^***^	-0.36578^***^	-0.35162^***^	-0.21434^***^	-0.21179^***^	-0.77629^***^	-0.84590^***^	-0.93323^***^	-0.97177^***^	-0.89608^***^
	*(0.04356)*	*(0.03642)*	*(0.03174)*	*(0.02028)*	*(0.01640)*	*(0.04657)*	*(0.03696)*	*(0.03200)*	*(0.02118)*	*(0.01636)*
Diff-in-Diff.:${\beta }_{2}$	0.38443^***^	0.16824^***^	-0.03536	-0.31558^***^	-0.39505^***^	-0.08471	-0.02684	0.02189	0.15957^***^	0.04818^**^
	*(0.05908)*	*(0.04993)*	*(0.04525)*	*(0.02879)*	*(0.02279)*	*(0.06358)*	*(0.05295)*	*(0.04604)*	*(0.02885)*	*(0.02437)*
*Adjusted R*^*2*^	*0.46687*	*0.45788*	*0.46379*	*0.43501*	*0.41684*	*0.52832*	*0.56028*	*0.57714*	*0.56163*	*0.52854*
*Commuting Background Pollution*	**MODEL 7**					**MODEL 8**				
Obs-vs-Pre:${\beta }_{1}$	-0.46123^***^	-0.40180^***^	-0.36146^***^	-0.21330^***^	-0.22006^***^	-0.91849^***^	-1.00777^***^	-1.14353^***^	-1.19544^***^	-1.10920^***^
	*(0.04397)*	*(0.03555)*	*(0.03049)*	*(0.01947)*	*(0.01574)*	*(0.05203)*	*(0.04033)*	*(0.03519)*	*(0.02199)*	*(0.01707)*
Diff-in-Diff.:${\beta }_{2}$	0.28294^***^	0.07384	-0.14840^***^	-0.48002^***^	-0.55120^***^	-0.07985	-0.00338	0.07716	0.23455^***^	0.12349^***^
	*(0.05747)*	*(0.04785)*	*(0.04327)*	*(0.02808)*	*(0.02224)*	*(0.06931)*	*(0.05650)*	*(0.04964)*	*(0.03021)*	*(0.02502)*
*Adjusted R*^*2*^	*0.49898*	*0.51861*	*0.52884*	*0.47958*	*0.46471*	*0.54462*	*0.57412*	*0.59319*	*0.59869*	*0.56455*
Observations	812	1,276	1,740	4,988	8,236	812	1,276	1,740	4,988	8,236
Intercept:${\alpha }_{0}$	Yes	Yes	Yes	Yes	Yes	Yes	Yes	Yes	Yes	Yes
Policy Dummy:${\beta }_{0}$	Yes	Yes	Yes	Yes	Yes	Yes	Yes	Yes	Yes	Yes
Meteorological Controls:${\beta }_{3}$	Yes	Yes	Yes	Yes	Yes	Yes	Yes	Yes	Yes	Yes
Weekend Dummy:${\beta }_{3}$	Yes	Yes	Yes	Yes	Yes	Yes	Yes	Yes	Yes	Yes

The dependent variable is the natural log of respective median pollution levels within the given time interval (full daily or commuting hours). Estimated impacts represent the marginal percent reduction in pollution concentration, and to measure the impact of moving from state 0 to state 1 in the treatment variables (${P}_{t}$, ${T}_{l}$ and $P_t\cdot T_l$), the change is estimated as $\%\Delta y=100\cdot\left(e^{\beta}-1\right)$.

One important consideration in the interpretation of magnitudes is the different timing of the lockdown and opening, and the cumulative dynamics of pollution. The opening period of interest, June 1, is over two months away from the lockdown period. While we choose non-overlapping time bands from which to explore these estimates, there are undoubtedly propagated effects from the lockdown which may be influencing the opening. Notably, the reduction estimates of ${\beta }_{1}$ are much larger around the opening given that by this time all areas had already shifted to concentration levels significantly lower than what would be predicted.

**Fig. 6:**
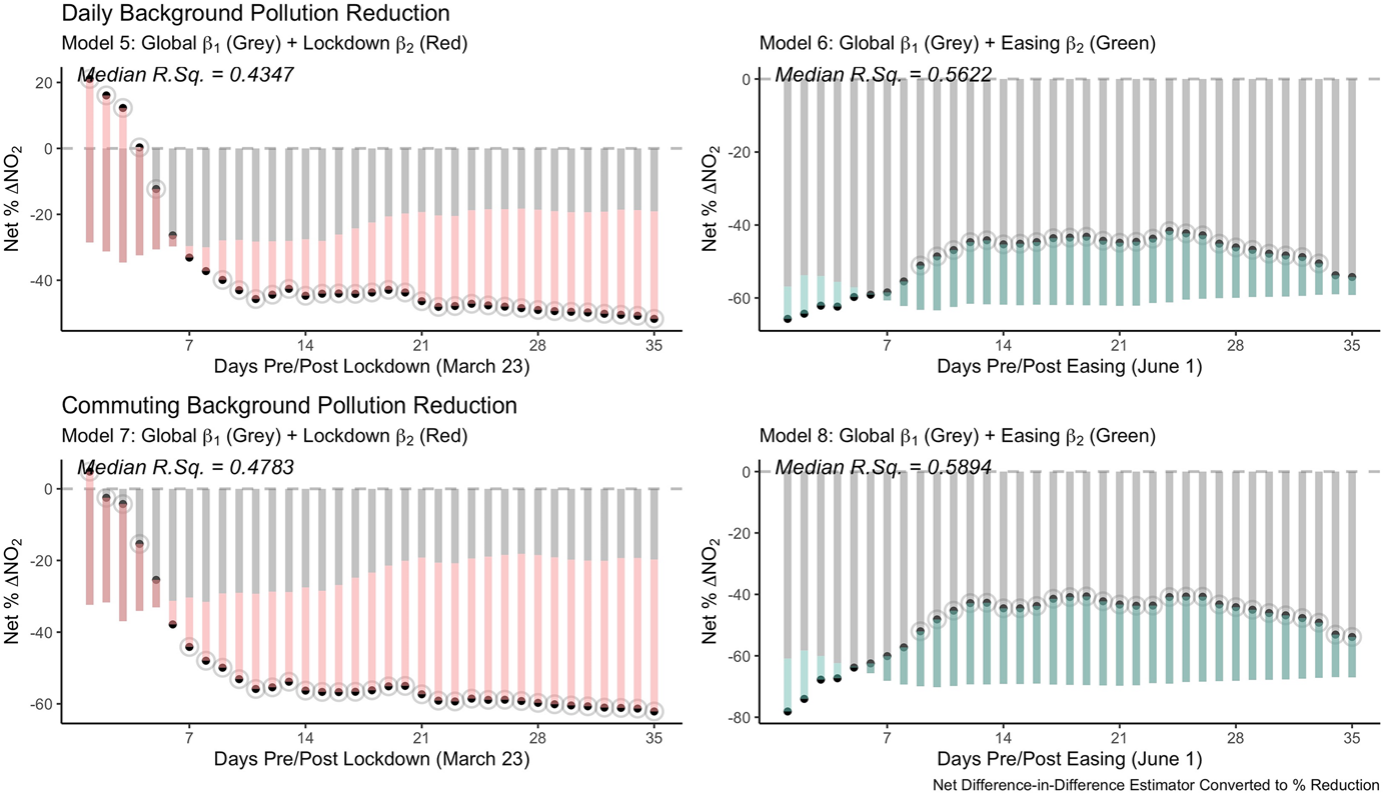
Difference-in-difference daily impact estimates (background pollution)

Figure 6 plots the daily adjustment to these estimated impacts for ambient level background pollution with traffic level results in Appendix Figure 10. These plots show the combined ${\beta }_{1}+{\beta }_{2}$ estimated percent change to concentrations up to five weeks following the respective announcement. Clear patterns emerge in considering the temporal signature of impacts in the days following either announcement. There is an adjustment period following the lockdown and easing announcements up to about a week following.

Overall, we observe clear global pollution reductions in comparing current trends with their expected predicted value. Both reductions around the date of the lockdown and the date of the easing reach between 40–50% in the weeks following, however breaking this down reveals the respective lockdown and easing specific components. During the lockdown period, we are able to observe some immediate increases in pollution in the subsequent days, falling to a reduction in concentration at about seven days. Following the easing however, we observe relative increases in pollution in the range of 17% (using the estimate of Model 6, week 3: $100\cdot\left(e^{0.1595}-1\right)=17.29$).

Comparing commuting hours with daily average estimates reveals the extent to which overall reductions were driven by key changes to normal working day signatures. Net pollution reductions following the first lockdown reaches the range of 50% for daily background pollution and up to 60% reduction specific to commuting hours.

As new workday patterns and transportation dynamics emerge, these results confirm the particular responsiveness of NO_2_ pollution to our daily activities. While there are overall global reductions in pollution levels compared to the expected patterns, both the lockdown and easing have additional interactive impacts in their own rights. As would be expected the short term impact of the lockdown was a reduction in pollution levels while the easing yielded relative increases.^13^

However, with areas having a different workforce composition adherence to home-working as well as to stay at home orders have varied spatially. By combining air pollution and Google Community Mobility Report data we estimated the impact on NO_2_ concentrations conditioned to different levels and types of mobility as such environmental imbalance may further propagate regional inequalities.

### Mobility-Conditioned Impacts

We use Google Mobility Reports for Local Authority daily mobility changes relative to pre-pandemic visits to six key categories: residential (measured as time spent at the location); retail and recreation amenities (non-essential); public transit amenities; workplaces; grocery and pharmacy (essential); and parks. Difference-in-difference impacts from Eq. 2 are estimated in turn on subsets of those areas with the highest mobility change and the lowest mobility change directly following the lockdown or easing.

For each of the six categories we focused on the percent change in mobility for the 2 weeks following the first lockdown (March 23, 2020) and the 2 weeks following the easing (June 1, 2020). From this, areas are ranked based on their respective mobility responsiveness to the intervention and split into above median and below median mobility groups. It is crucial to interpret the estimated responsiveness in terms of this median value to determine what above and below this value represent.

Following the first lockdown, we observed a 26% median increase in residential mobility compared to decreases in the other categories. The largest median reductions in mobility following the first lockdown occurred in changes to workplaces (-63%), retail (-74%) and transport (-64%). Smaller reductions were observed around parks (-21%), and grocery shops (-30%).

Following the easing on June 1, 2020, mobility around residence was lower (19%) and visits to parks increased compared to pre-lockdown levels to 26%. However, the median percentage change remained negative for all the other categories but with different magnitudes (workplaces -49%, transport -44%, retail -58%, grocery and pharmacy -15%).

**Fig. 7:**
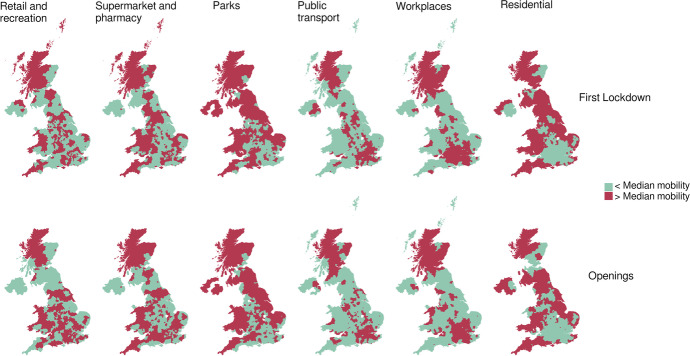
Level of human mobility in the UK by Google type of place visited categorised as above and below median national values 2 weeks after the First Lockdown (March 23, 2020) and the Openings (June 1, 2020)

Figure 7 shows which areas fall above and below the median level of mobility for each Google category. Overall, we see that the ability to comply with mobility restrictions during the first lockdown and the reaction to the openings varies significantly across space. The spatial variations in areas characterised by mobility levels above or below the median values do not change significantly in the two time periods considered. This suggests that areas which were less able to adhere to mobility restrictions were also those that more quickly tended to return to relatively higher levels of mobility after the re-openings and viceversa.

Higher values of residential mobility are very much concentrated in and around Greater London indicating a wider capacity to comply with the stay at home order during the lockdown but also to keep shielding after the openings. As expected, visits to the workplace show the opposite spatial pattern, being below the median value mostly where residential mobility is above, and this provides a quite clear picture of the prevalence of home-working in the south of England and Scotland. Along with less workplace visits some of these areas also see lower use of public transit. Visits to parks tended to be more frequent in southern parts of the UK and, particularly after the openings in summer time, in coastal areas. For the categories of retail and grocery we do not see any specific spatial patterns as areas above and below the median values seem to be randomly distributed throughout the country.

By classifying areas based on the median mobility change, we can then further decompose our estimated reductions in NO_2_ concentration conditioned to the varying ability to reduce the number of visits to different places.

#### Estimated NO_2_ Concentration Reductions Following the First Lockdown (March 23)

**Fig. 8:**
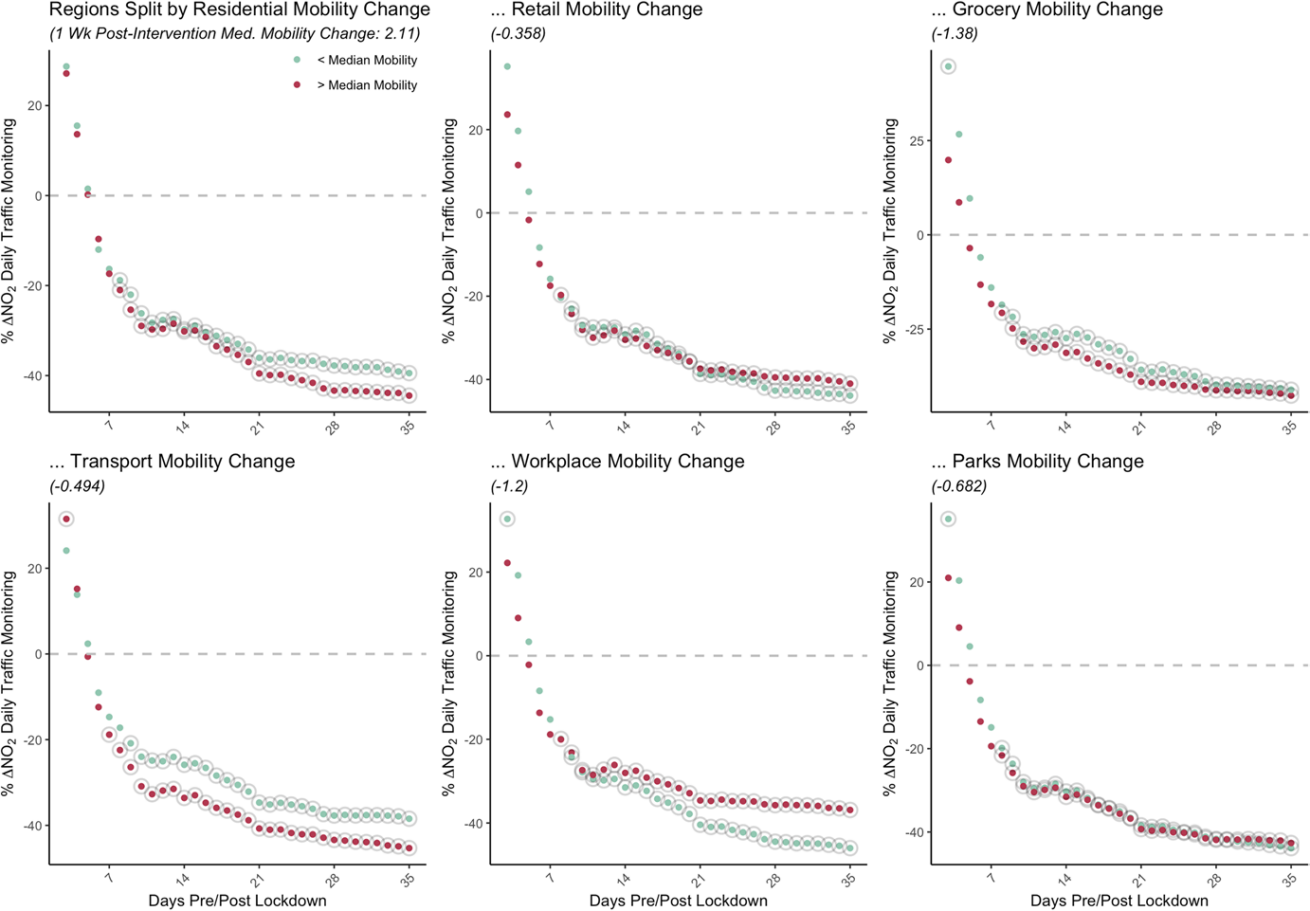
Mobility-conditioned lockdown impacts (daily traffic monitoring)

Figure 8 shows the estimated marginal difference-in-difference impact of the lockdown effect ${\beta }_{2}$. In splitting areas into above and below median post-lockdown mobility, we observe differences in pollution reductions after about two weeks from locations with variation in residential, transport and workplace mobility changes. For areas which had above median residential mobility change, indicating more time spent around residences, stronger pollution reductions are observed compared to areas with relatively less time spent at home.

Comparatively, similar deviations are observed with mobility around public transit and workplaces. Where locations had a below median change in mobility around public transit, they had a stronger reduction in movement around these amenities. For this grouping of areas, we observe weaker pollution reductions relative to those areas which have less of a reduction in transit-based mobility. With residents using public transit less, many shifted transportation mode towards private cars with a potential to counteract some of the general concentration reductions post-lockdown. While overall changes to concentrations are negative, this gap in reduction signals the importance that transportation modes and changing patterns have in determining these dynamics.

Along similar lines, we observe the greatest reduction in concentration for locations which had higher reduction in mobility around workplaces. In a mirror to the increased residential mobility, post-lockdown trends see massive decreases in workplace mobility. As residential mobility increased, and workplace mobility decreased, NO2 concentration changes appear intimately linked to the degree with which human patterns change.

While we see some divergence of impacts among traffic-based monitoring stations for amenities associated with urban cores such as public transport infrastructure and retail, there is a notably significant difference in background ambient pollution reductions conditional on mobility around parks and greenspace amenities (Appendix Figure 11). Where areas have strong reductions in mobility changes around parks in the week following the lockdown (below the median -21%), there are stronger reductions in background pollution. This effect is not discernible with traffic monitoring stations. This is likely due to the location of traffic stations which tend to be near major city junctions and therefore, generally further from public parks, making such stations less sensitive to these visits.

#### Estimated NO_2_ Concentration Reductions Following Easing (June 1)

**Fig. 9:**
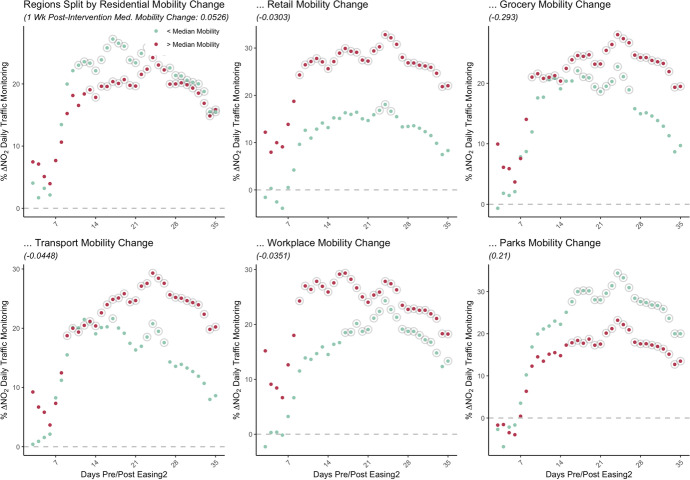
Mobility-conditioned easing impacts (daily traffic monitoring)

Patterns suggest that post-easing, areas which reduced their mobility around residences, and thus travelled by some means to other destinations, had some stronger increases in concentration in the short term. Overall, however, all areas experienced increases in pollution following the easing restrictions on June 1, 2020 (Fig. 9).

The most significant difference stems from considering areas which had positive changes to retail, transport and workplace visits compared to those which had reductions following the easing. The post-easing increase in NO_2_ concentrations appears, in fact, primarily driven by locations which had increased mobility around all these key categories of urban life. During the easing period, residents increasingly moved back to the workplace and shopping areas and this  is linked with significant pollution increases for those areas more positively reacting to the easing (with mobility above the median) compared to those which did not immediately respond to the easing through increased mobility. On the contrary, areas where residential and parks mobility were above average show a less substantial increase in concentration of Nitrogen Dioxide. 

## Discussion and Conclusion

While we saw overall reductions in nitrogen-based concentrations following lockdowns around the world and in the UK due to lower levels of human mobility, we know that parts of the population have different propensities and abilities to comply with stay-at-home orders. As population segments, in different locations, begin returning to pre-pandemic mobility levels at different times and in stages, the impact of our patterns on pollution concentration is bound to be complex. Viewing the responsiveness of concentration to the human mobility breakdown for different purposes is key for understanding the implications of transitioning forward.

### Main Findings and Interpretation

By decomposing lockdown and easing pollution impacts into hourly breakdowns our results show a clear difference in reductions during typical commuting hours. Our estimates show that, following the lockdown, reductions in NO_2_ concentration during typical commuting hours were systematically larger than daily average reductions. With the reopening of businesses and part of the population returning to their workplaces we see pollution increasing, albeit with commuting hour dynamics having less relative impact. 

Leveraging Google Mobility Reports we further decompose these impacts accounting for local variations in the compliance with lockdown mobility restrictions. We can identify clear spatial patterns specifically in relation to mobility around workplaces, public transit, residence and parks, with people living in Greater London and in some parts of the South of England and Scotland showing a relatively higher propensity to stay at home. Following the lockdown these areas have benefitted from a larger reduction in NO_2_ concentration given the increased ability to work at home and stay around the residence. However, this is partly counterbalanced by lower reductions in NO_2_ due to less usage of public transport which might correspond to some people turning to driving. Given the high propensity in home-working characterising these areas, we can hypothesise car-drivers travelling mostly non-commuting trips. Such results seem to confirm that teleworkers might be more willing to travel longer distances with private means, reducing the potential benefit in air quality of less commuting (Bunditz and Tranos 2020).

With the easing of the restrictions and re-opening of the economy, visits to non-essential shops and workplaces brought about a higher increase in NO_2_ concentration levels. Even at this stage we see commuting as a key component in nitrogen-based pollution with areas where higher movements around residences are more likely to have weaker NO_2_ concentration increases. In the context of a general increase in mobility, however, areas more apt to use public transit did not gain any benefit in terms of NO_2_ concentration levels, which, on the contrary, seems to be signalling more mobility overall.

### Challenges and Future Works

As we rely on Google Mobility Reports, it is important to underline some of the challenges characterising these data. Firstly, the geographic scale at which measures are available represents Local Authority averages which may include both urban and rural areas. This limits the granularity with which any small-area variation can be used to fine-tune the mobility-conditioned impacts. Further, pure count and visit numbers are not available and thus our thresholds from which we classify large or weak mobility changes is based on the overall index of mobility provided and we do not have detailed information on mode of travel. Future work should explore other sources of mobility data at higher spatial and temporal resolution allowing for the possibility to detect travel modes (Xiao et al., 2015) to better uncover links between workday commuting choices and pollution. At the same time, we note that the robustness of the direction of impacts across a range of model specifications in our aggregate results highlights significant co-movements deserving of consideration in the interim period forward.

Lastly, Google mobility data are crowd-sourced and as such are not the result of random sampling of a selected population, but representative of a proportion of Google users who have their location history available. While it is important to acknowledge this limitation, the generally large level of Google users and the temporal resolution of both pollution and mobility data enable us to rely on the general trends seen in this analysis. Opportunities to validate these data may emerge by combining Google Mobility national aggregate variations with changes in the average trips by purpose of travel captured by the National Travel Survey which will be the object of future work.

As different regions have different propensities and capacity to adhere to restrictions, whether through the type of workforce and industry prevalent, the demographic composition, or public transportation infrastructure, our average estimated impacts mask a variation whereby some locations may be additionally burdened by emission reduction inequality. Additional analysis, combining more auxiliary data, will be carried out to uncover such contextual drivers of change in urban pollution concentration as well as evaluating how these relationships interact with other pollutants.

### Policy Suggestions

Although there are a number of complex ecological and environmental interactions which come into play, through an exploratory analysis and modelling exercise we observe systematic relationships between periods of lockdown and openings, traditional workday temporal signatures, mobility patterns and NO_2_ concentration reductions. As new restrictions and openings continued through to the end of the COVID-19 pandemic, there are significant environmental consequences to the re-implementation and easing of mobility restrictions. Moreover, the short term shift towards more home-based work, while temporary, may significantly reshape many industries under the parameters of their respective workforces being able to efficiently conduct their jobs from home.

As financially poorer people are often employed in occupations that do not provide opportunities to work from home (Patel et al., 2020), decomposing the signature of pollutants commonly attributed to combustion emissions and understanding its responsiveness to human behaviour is the first step to understand how transitioning towards potentially new working norms could exacerbate existing inequality from an environmental perspective. In light of this, it is important for policy makers to target areas with a workforce composition that has lower propensity to home-working and ensure that sustainable travel modes are available for commuting.

This could be accomplished by investment in cycling and walking infrastructure as well as pressure for a capillary bus network.

Overall, our study provides evidence of the potential benefit of home-working in reducing concentration of NO_2_. While more contextual variables are needed to assess the advantages and disadvantages of home-working more broadly, it can be considered as part of the policy toolkit to reduce nitrogen-based concentrations. At the same time, as we note that some of the areas with more home-working also had less usage of public transport, which in the lockdown period was linked to lower NO_2_ drops, we could hypothesise more people turning to cars and potentially having longer distance trips as suggested in Bunditz and Tranos (2020). As a consequence, to maximise the possible benefit of home-working, making sure that high quality services are accessible in the proximity of people’s places of residence becomes critical.

## Figures and Tables

**Fig. 10:**
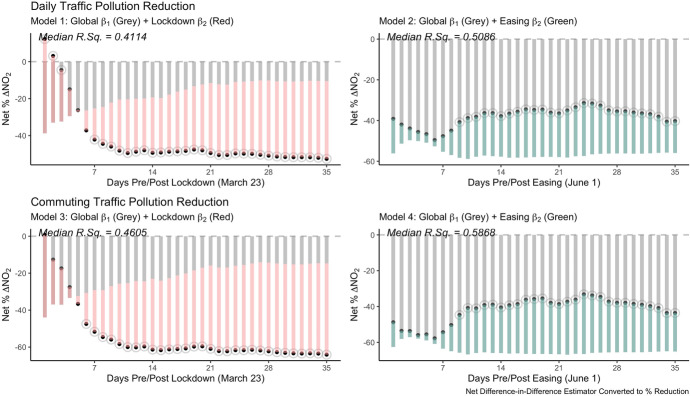
Difference-in-difference daily impact estimates (traffic pollution)

**Fig. 11:**
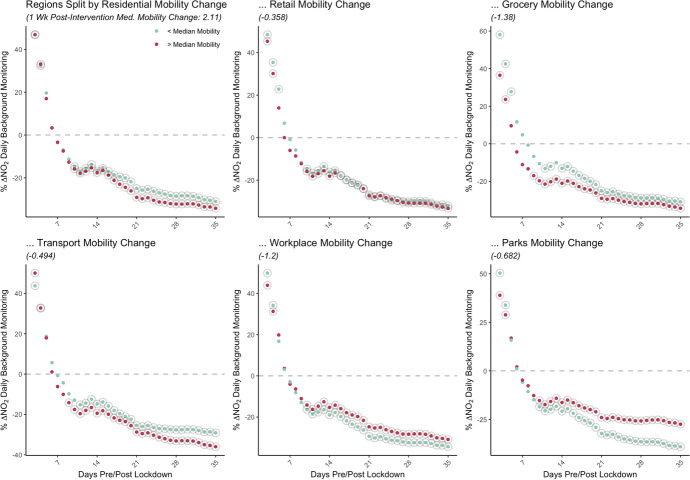
Mobility-conditioned lockdown impacts (daily background monitoring)

## Footnotes

1 Prime Minister’s statement on COVID-19: 23 March 2020: https://www.gov.uk/government/speeches/pm-address-to-the-nation-on-coronavirus-23-march-2020

2 Pollution data is extracted using the *openair v0.3–8* library in ***R*** for all days from January 1, 2015 to September 20, 2020.

3 Statistical tests and visual inspection between the respective series in the same LAD are run before being combined. Tests on granger causality in both directions suggest that series are significantly similar and co-move together with high correlations all over 0.7 between them.

4 The median value is preferred so as not to be influenced by outliers in a respective time period.

5 When missing, daily values are iteratively filled with the rolling average of the previous seven days of pollution to generate a balanced time series.

6 Mobility data from google is scaled to Local Authority boundaries covering all the UK. The delineation and spatial merging of mobility data to Local Authority boundaries was facilitated greatly by the Data Science Campus: https://github.com/datasciencecampus/google-mobility-reports-data

7 During this time in the UK, there was little variation among localities in terms of the announcements. While the Fall of 2020 is marked by more local and regional lockdowns and openings, those occurring over the summer were targeted at the nation.

8 https://www.instituteforgovernment.org.uk/charts/uk-government-coronavirus-lockdowns

9 https://www.health.org.uk/news-and-comment/charts-and-infographics/covid-19-policy-tracker

10 Trends are plotted at the national level and built off the aggregation of individual monitoring station predictions. Individual parallel trend assumptions for each monitoring station available upon request.

11 Estimated impacts on all easing announcements show similar effects, the most statistically significant occurring towards the start of the easings and decaying towards the later as cumulative openings increased mobility. We present results from the second of the openings where estimated results indicate the largest change in pollution. Results for all easings available upon request.

12 Full estimated results and diagnostics available upon request.

13 Other localities show similar trends regarding a general reduction and flattening of the hourly average NO_2_ distribution – although there are differences in the overall concentration of NO_2_ which is independent from the COVID-19 related circumstances. All plots are available upon request.
